# *SCN2A*-Related Epilepsy: The Phenotypic Spectrum, Treatment and Prognosis

**DOI:** 10.3389/fnmol.2022.809951

**Published:** 2022-03-30

**Authors:** Qi Zeng, Ying Yang, Jing Duan, Xueyang Niu, Yi Chen, Dan Wang, Jing Zhang, Jiaoyang Chen, Xiaoling Yang, Jinliang Li, Zhixian Yang, Yuwu Jiang, Jianxiang Liao, Yuehua Zhang

**Affiliations:** ^1^Department of Pediatrics, Peking University First Hospital, Beijing, China; ^2^Department of Neurology, Shenzhen Children’s Hospital, Shenzhen, China

**Keywords:** epilepsy, *SCN2A* gene, variant, phenotype, treatment

## Abstract

**Objective:**

The aim of this study was to analyze the phenotypic spectrum, treatment, and prognosis of 72 Chinese children with *SCN2A* variants.

**Methods:**

The *SCN2A* variants were detected by next-generation sequencing. All patients were followed up at a pediatric neurology clinic in our hospital or by telephone.

**Results:**

In 72 patients with *SCN2A* variants, the seizure onset age ranged from the first day of life to 2 years and 6 months. The epilepsy phenotypes included febrile seizures (plus) (*n* = 2), benign (familial) infantile epilepsy (*n* = 9), benign familial neonatal-infantile epilepsy (*n* = 3), benign neonatal epilepsy (*n* = 1), West syndrome (*n* = 16), Ohtahara syndrome (*n* = 15), epilepsy of infancy with migrating focal seizures (*n* = 2), Dravet syndrome (*n* = 1), early infantile epileptic encephalopathy (*n* = 15), and unclassifiable developmental and epileptic encephalopathy (*n* = 8). Approximately 79.2% (57/72) patients had varying degrees of developmental delay. All patients had abnormal MRI findings with developmental delay. 91.7% (55/60) patients with *de novo SCN2A* variants had development delay, while only 16.7% (2/12) patients with inherited *SCN2A* variants had abnormal development. 83.9% (26/31) *SCN2A* variants that were located in transmembrane regions of the protein were detected in patients with development delay. Approximately 69.2% (9/13) *SCN2A* variants detected in patients with normal development were located in the non-transmembrane regions. Approximately 54.2% (39/72) patients were seizure-free at a median age of 8 months. Oxcarbazepine has been used by 38 patients, and seizure-free was observed in 11 of them (11/38, 28.9%), while 6 patients had seizure worsening by oxcarbazepine. All 3 patients used oxcarbazepine and with seizure onset age > 1 year presented seizure exacerbation after taking oxcarbazepine. Valproate has been used by 53 patients, seizure-free was observed in 22.6% (12/53) of them.

**Conclusion:**

The phenotypic spectrum of *SCN2A*-related epilepsy was broad, ranging from benign epilepsy in neonate and infancy to severe epileptic encephalopathy. Oxcarbazepine and valproate were the most effective drugs in epilepsy patients with *SCN2A* variants. Sodium channel blockers often worsen seizures in patients with seizure onset beyond 1 year of age. Abnormal brain MRI findings and *de novo* variations were often related to poor prognosis. Most *SCN2A* variants located in transmembrane regions were related to patients with developmental delay.

## Introduction

The etiology of epilepsy is a major determinant of clinical course and prognosis. Six etiologic groups of epilepsy include structural, metabolic, genetic, infectious, and immune, as well as an unknown group (Scheffer et al., 2017). As genetic testing is broadly used in pediatric neurology, more than half of epilepsy children are thought to have a genetic cause (Reif et al., 2017). At present, voltage-gated sodium channel genes such as *SCN1A*, *SCN2A*, *SCN3A*, and *SCN8A* were reported to be causative genes of epilepsy (Ademuwagun et al., 2021), among them *SCN2A* has been reported to be the second most common, next only to *SCN1A*, the first reported causative gene for epilepsy (Heyne et al., 2019). Epilepsy caused by *SCN2A* variants mostly starts in early childhood and has a wide phenotypic spectrum, ranging from self-limited epilepsy with a favorable outcome to developmental and epileptic encephalopathy, and most of them respond well to sodium channel blockers (SCBs) (Grinton et al., 2015; Trump et al., 2016; Dilena et al., 2017; Flor-Hirsch et al., 2018; Kim et al., 2020; Melikishvili et al., 2020; Miao et al., 2020; Penkl et al., 2021). China has a large population and a large number of epilepsy children. However, the epilepsy phenotypes and prognosis caused by *SCN2A* variation in Chinese children have not yet been studied in a large sample. In this study, the phenotypic spectrum, treatment, and prognosis of epilepsy children with *SCN2A* variants were studied in a Chinese cohort from two pediatric clinical centers.

## Materials and Methods

### Participants

In this study, epilepsy children who were suspected of genetic etiology and identified with *SCN2A* variants by next-generation sequencing were enrolled in Peking University First Hospital and Shenzhen Children’s hospital from September 2006 to January 2021. All epilepsy patients fulfilled the following criteria: (1) no identifiable immediate or remote cause and (2) no metabolic or mitochondrial disorders. Clinical information includes the age of seizure onset, seizure types, developmental milestones, neurologic status, electroencephalogram (EEG), brain MRI, and treatment data of the patients and their relatives were collected using a pre-test questionnaire completed by the recruiting clinician by telephone or from medical records. Patients were followed up at a pediatric neurology clinic at our hospital or by telephone. The effect of anti-seizure medication (ASM) therapy were retrospectively assessed and classified according to the judgment of the treating physicians into seizure freedom, seizure reduction (reduction in seizure frequency > 50%), no effect or seizure worsening. This study was approved by the Ethics Committee of Peking University First Hospital and Shenzhen Children’s hospital, respectively. The written informed consent for the analysis and publication of clinical and genetic details was obtained from the patients or their parents.

### Genetic Analysis

Blood samples were obtained from these probands and their family members when possible. Genomic DNA was extracted from peripheral blood by a standard method. All patients were screened for pathogenic variants either through a custom-designed gene panel in which candidate genes associated with epilepsy including *SCN2A* was selected as the genes of interest or by whole-exome sequencing. The potential pathogenic variations suggested by the targeted next-generation sequencing were validated using Sanger sequencing.

## Results

### *SCN2A* Variants

**FIGURE 1:**
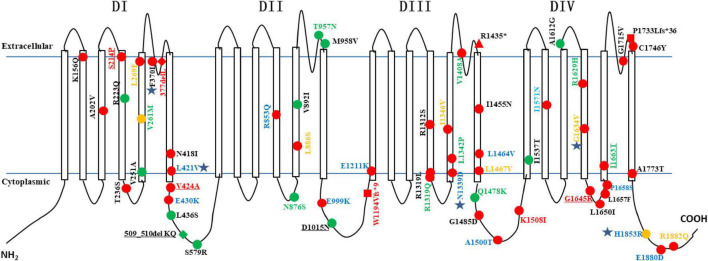
The developmental outcome and treatment effects to oxcarbazepine of epilepsy patients with *SCN2A* variants. A total of 59 *SCN2A* variants was included. The shapes of variation sites represent different variation types (circle = missense variation; triangle = nonsense variation; square = frameshift variation; rhombu = in-frame deletion variation). The colors of the shape represent different developmental outcome (red = developmental delay; green = normal development; orange = both patients with normal development and developmental delay were observed). The colors of the variants represent the treatment effects of oxcarbazepine (green = seizure freedom; blue = seizure reduction; orange = no effect; red = seizure worsening; black = never used). The variation underlined indicates fever-sensitivity. Pentagons indicate one patient with the variants died.

A total of 72 unrelated epilepsy patients with heterozygous *SCN2A* variants were collected. Among them, patients 1–8 have been reported in a previous study of benign familial epilepsy (Zeng et al., 2018). Fifty-nine *SCN2A* variants were identified, including 54 missense variants (91.5%, 54/59), 2 frameshift variants, 2 in-frame deletion variants, and 1 non-sense variant. A total of 22 *SCN2A* variants were novel. The *SCN2A* variants were scattered in different regions of the gene, and there were no obvious hot spot variants (see Figure 1). V261M, R853Q, H1853R, E999K, E1211K, R1319Q, A1500T, R1629H, and P1658S were recurrent variants, each was identified in two or three patients (see Table l). A total of 12 (12/72, 16.7%) patients had inherited variants, and the other 60 (60/72, 83.3%) patients had *de novo* variants. All 12 patients with inherited variants had a family history of epilepsy or febrile seizures. All of the affected parents had heterozygous variants as their children, except the mother of patient 48. She carries the same *SCN2A* variant with a ratio of about 21.5% in the peripheral blood by next-generation sequencing.

**TABLE 1:** The genetic testing results and clinical features of 72 patients with *SCN2A* variants.

Patient	Gender	Variation	Reported/ novel	Inheritance	Seizure onset age	Age at last follow-up	EEG features	Seizure types	MRI	Psychomotor development	Phenotype	Age at seizure free	Seizure free therapy	Other conditions
1	Male	c.2627A > G(p.N876S)	Reported	Maternal	2 months	5 years 6 months	Normal	FS	Normal	Normal	BFNIE	4 months	OXC	
2	Female	c.2674G > A(p.V892I)	Reported	Maternal	2 male	6 years 11 months	FS	FS	Normal	Normal	BFNIE	6 months	LEV, TPM	
3	Female	c.2872A > G(p.M958V)	Reported	Paternal	3 months	6 years 1 months	FD	FS	Normal	Normal	BFNIE	4 months	VPA	
4	Male	c.668G > A(p.R223Q)	Reported	Paternal	4 months	11 years	Normal	FS	Normal	Normal	BFIE	7 months	VPA	
5	Male	c.752T > C(p.V251A)	Reported	Maternal	3 months	9 years	FD	FS	Normal	Normal	BFIE	4 months	PB	
6	Male	c.1307T > C(p.L436S)	Reported	Maternal	3 months	10 years	Normal	FS	Normal	Normal	BFIE	8 months	VPA	
7	Female	c.1737C > G(p.S579R)	Reported	Paternal	1 year 2 months	6 years 2 months	Normal	FS	Normal	Normal	BFIE	1 year 3 months	Self-limited	
8	Male	c.4835C > G(p.A1612G)	Reported	Maternal	3 months	8 years 10 months	Normal	FS	Normal	Normal	BFIE	4 months	Self-limited	
9	Male	c.1523_1528 delAGAAAC (p.509_510del KQ)	Novel	Paternal	11 months	10 years	GD	GTCS, AtS	Normal	Normal	FSP	2 years	VPA	Fever sensetivity
10	Male	c.4988T > C(p.I1663T)	Novel	Maternal	6 months	7 years	GD, FD, MS	GTCS, MS, FS	Normal	Delay, walk at 3 years 6 months, speak at 1 year 3 months	DS	5 years	VPA, CLZ, OXC	Fever sensetivity
11	Male	c.1108T > C(p.F370L)	Novel	*De novo*	8 days	2 years 7 months	BS, FS, SS	TSS, SS, FS	Normal	Delay, cannot control head and speak	OS	8 months	VGB, TPM	Died at 2 years 7 months
12	Male	c.781G > A(p.V261M)	Reported	*De novo*	4 days	5 years	MD, FS	SS, FS	Normal	Delay, head control at 2 years 6 months, sit alone at 4 years	EIEE	8 months	VPA, TPM	
13	Female	c.466A > C(p.K156Q)	Novel	*De novo*	3 days	1 year 4 months	GD, TS	TS	DWMM	Delay, cannot control head and speak	EIEE	Non-remission		
14	Female	c.1261T > G(p.L421V)	Reported	*De novo*	10 days	4 years 3 months	BS, HPS, TSS, FS, SS	TSS, SS, FS	ACC, DWMM, DFTL, ELV	Delay, cannot control head and speak	OS → WS	Non-remission		Died at 4 years 3 months
15	Male	c.5558A > G(p.H1853R)	Reported	*De novo*	2 days	2 years 9 months	BS, HPS, FS, SS	TSS, SS, FS	Normal	Delay, cannot control head and speak	OS → WS	8 months	TPM	Died at 2 years 9 months
16	Male	c.4223T > C(p.V1408A)	Reported	*De novo*	2 days	8 years 1 months	FD	FS	Normal	Delay, walk at 1 year 8 months, normal speech	EIEE	5 months	OXC, LEV	
17	Male	c.2995G > A(p.E999K)	Reported	*De novo*	2 days	3 years 10 months	HPS, MD	SS, FS	Normal	Delay, cannot control head and speak	WS	3 months	VPA, LEV, TPM	
18	Male	c.4364T > A(p.I1455N)	Reported	*De novo*	2 days	7 years	HPS, FS, SS	SS, FS	Normal	Delay, cannot control head and speak	WS	2 years 5 months	TPM, VPA	
19	Male	c.4454G > A(p. G1485D)	Novel	*De novo*	3 months	7 years 6 months	Normal	FS	Normal	Delay, walk at 1 years 4 months, poor school performance	EIEE	2 years	VPA	
20	Male	c.1271T > C(p. V424A)	Reported	*De novo*	1 days	8 years 2 months	GD, FS	FS, GTCS	DFTL, ELV	Delay, cannot control head and speak	EIEE	Non-remission		Fever sensetivity
21	Female	c.5144G > T(p. G1715V)	Reported	*De novo*	8 months	8 years 3 months	HPS, FD, MD, AS	SS, AS	Normal	Delay, walk at 2 years 1 months, can only speak a few words	WS	3 years 1 months	VPA, LTG	
22	Male	c.3631G > A(p E1211K)	Reported	*De novo*	4 months	6 years 11 months	HPS, FD, SS, FS	SS, FS	ELV	Delay, control head at 1 years 4 months, cannot sit alone and speak	WS	Non-remission		
23	Male	c.4498G > A(p.A1500T)	Reported	*De novo*	2 days	1 years 6 months	HPS, FD, SS, FS	SS, FS, TSS	Normal	Delay, cannot control head and speak	WS	Non-remission		
24	Male	c.5196delC (p.P1733Lfs*36)	Novel	*De novo*	11 months	5 years	HPS, SS	SS	DFTL	Delay, cannot control head and speak	WS	Non-remission		
25	Male	c.4933G > A(p.G1645R)	Novel	*De novo*	2 years	5 years 11 months	GD	GTCS, FS	Normal	Delay, poor speech and school performance	DEE	5 years 4 months	LEV, VPA	Fever sensetivity
26	Female	c.4399C > G(p.L1467V)	Novel	*De novo*	10 months	6 years 7 months	FD	FS	ELV	Delay before seizure onset, cannot walk and speak	DEE	1 years 11 months	VPA, LEV, TPM	
27	Male	c.1128_1130de lCTT(p.377del L)	Novel	*De novo*	2 years 3 months	6 years 4 months	FD	FS	Normal	Delay before seizure onset, walk at 1 years 6 months, cannot speak	DEE	2 years 10 months	VPA, LEV	
28	Male	c.4303C > T(p.R1435*)	Reported	*De novo*	2 years 6 months	7 years 3 months	FD	FS	Normal	Delay before seizure onset, walk at 2 years, cannot speak	DEE	3 years 4 months	VPA, LEV, TPM	ASD
29	Female	c.4015A > G(p.N1339D)	Reported	*De novo*	14 days	2 years 8 months	BS, HPS, FS, SS, MS	TSS, FS, MS	ELV, ACC, DFTL	Delay, cannot control head and speak	OS → WS	Non-remission		Died at 2 years 8 months
30	Male	c.781G > A(p.V261M)	Reported	*De novo*	3 days	4 years 1 months	MD, FS	FS	Normal	Delay, walk at 1 year 2 months, slightly poor language performance	EIEE	Non-remission		
31	Male	c.5558A > G(p.H1853R)	Reported	*De novo*	2 days	5 years 8 months	FD, FS	FS	Normal	Delay, walk at 1 year 6 months, poor language performance	EIEE	1 month	PB	
32	Male	c.605C > T(p.A202V)	Reported	*De novo*	3 days	6 years 2 months	MD, FS	FS	Normal	Delay, walk at 1 year 2 months, slightly poor language performance	EIEE	3 months	PB	
33	Male	c.5317G > A(p.A1773T)	Reported	*De novo*	8 months	13 years	HPS, GD, FD, SS, MS, AS	FS, SS, MS, AS	Normal	Delay, walk at 4 years, cannot speak	WS	Non-remission		
34	Male	c.3631G > A(p.E1211K)	Reported	*De novo*	1 year	5 years	HPS, MD, SS	SS	DFL, ELV	Delay before seizure onset, walk at 4 years 8 months, cannot speak	WS	Non-remission		ASD
35	Male	c.3956G > T(p.R1319L)	Reported	*De novo*	2 days	6 years 1 month	MD, FD, FS, SS, AS	FS, SS, AS	Normal	Delay, walk with help at 6 years, cannot speak	EIEE	Non-remission		
36	Male	c.4712T > C(p.I1571T)	Reported	*De novo*	2 days	1 year 3 months	BS, HPS, MD, FS, SS	TSS, FS, S	Normal	Delay, cannot control head and speak	OS → WS	Non-remission		
37	Female	c.4523A > T(p.K1508I)	Novel	*De novo*	1 day	2 years 9 months	BS, HPS, MD, FS, SS, TS	TS, SS, FS	DFTL, DWMM	Delay, cannot control head and speak	OS → WS	Non-remission		
38	Female	c.4498G > A(p.A1500T	Reported	*De novo*	2 days	3 years 9 months	BS, HPS, FD, FS, SS	TSS, SS, FS	Normal	Delay, cannot control head and speak	OS → WS	Non-remission		
39	Male	c.4025T > C(p.L1342P)	Reported	*De novo*	6 months	4 years 5 months	HPS, MD	SS	ACC, DWMM, ELV	Delay, cannot control head and speak	WS	1 year 6 months	VPA, OXC	
40	Male	c.4036A > G(p.I1346V)	Reported	*De novo*	1 days	3 years 11 months	BS, HPS, FS, SS, TSS	FS, SS, TSS	ACC, DFTL	Delay, cannot control head and speak	OS → WS	Non-remission		
41	Male	c.807G > T(p.L269F)	Novel	*De novo*	1 days	2 year 5 months	HPS, MD, FS, SS	FS, SS	DFTL	Delay, cannot control head and speak	WS	3 months	LEV	
42	Male	c.4972C > T(p.P1658S)	Reported	*De novo*	1 days	3 years 3 months	MD, FD	FS	DFTL, HA	Delay, cannot control head and speak	EIEE	Non-remission		
43	Male	c.4948C > A(p.L1650I)	Novel	*De novo*	2 days	1 year	BS, HPS, FS, SS	FS, SS, TSS	Normal	Delay, cannot control head and speak	OS → WS	Non-remission		
44	Male	c.5237G > A(p.C1746Y)	Novel	*De novo*	1 year 3 months	6 years 9 months	GD, MD, FD, SS	FS, SS	ELV	Delay before seizure onset, cannot walk and speak	EIEE	1 year 10 months	LEV, VPA, TPM	
45	Female	c.2657T > C(p.L886S)	Reported	*De novo*	1 days	5 years 1 months	BS, FD, FS	TSS, FS	DWMM	Delay, cannot control head and speak	OS	Non-remission		
46	Female	c.4432C > A(p.Q1478K)	Novel	*De novo*	1 month (30 days)	3 years 3 months	FS	FS	Normal	Normal	BIE	3 months	OXC	
47	Female	c.3579_3580 delCT > (p.W1194Vfs*9)	Reported	*De novo*	1 year 5 months	5 year	GD, MD, FD, SS	SS	HA	Delay before seizure onset, cannot walk and speak	DEE	Non-remission		
48	Female	c.2558G > A(p.R853Q)	Reported	Maternal	10 months	1 year 2 months	HPS, GD, MD, FS	FS, SS	DFTL,ACC	Delay before seizure onset, cannot sit and speak	WS	Non-remission		
49	Male	c.781G > A(p.V261M)	Reported	*De novo*	2 days	2 years 3 months	Normal	FS	Normal	Normal	BNE	3 months	OXC	
50	Male	c.640T > C(p.S214P)	Reported	*De novo*	2 months	2 years 11 months	HPS, FD, SS	FS, SS	ACC	Delay, cannot control head and speak	WS	Non-remission		Fever sensetivity
51	Female	c.3936G > T(p.R1312S)	Novel	*De novo*	4 months	1 year 6 months	MD, FS, SS	FS, SS	DFTL	Delay before seizure onset, cannot control head and speak	DEE	Non-remission		
52	Male	c.5558A > G(p.H1853R)	Reported	*De novo*	9 days	1 year 8 months	BS, HPS, FD, FS, SS, TSS	TSS, SS, FS	ACC, DWMM, ELV	Delay, cannot control head and speak	OS → WS	Non-remission		
53	Male	c.5640A > C(p.E1880D)	Novel	*De novo*	1 days	1 year 4 months	HPS, ED, MD, SS, MS, TS	TS, FS, SS, MS	DFTL	Delay, cannot control head and speak	WS	Non-remission		
54	Female	c.1253A > T(p.N418I)	Novel	*De novo*	1 year 5 months	2 years 3 months	MD, SS, MS	FS, MS, SS	Normal	Delay before seizure onset, cannot walk and speak	DEE	Non-remission		
55	Female	c.3043G > A(p.D1015N)	Reported	*De novo*	1 year	13 years	Normal	FS	Normal	Normal	FSs	11 years	VPA	Fever sensetivity
56	Female	c.4886G > A(p.R1629H)	Reported	*De novo*	1 days	5 years 2 months	BS, MD	TSS, FS	Normal	Delay, walk at 2 years, can speak only a few words	OS	4 m	OXC, KD	
57	Male	c.3956G > A(p.R1319Q)	Reported	*De novo*	4 months	2 years 2 months	MD, FS	FS	Normal	Normal	BIE	5 months	OXC	
58	Male	c.2558G > A(p.R853Q)	Reported	*De novo*	11 months	1 years	HPS, FD, SS	SS, FS	ELV, DFTL	Delay before seizure onset, 6 months control head	WS	Non-remission		
59	Male	c.4886G > A(p.R1629H)	Reported	*De novo*	4 days	5 years 11 months	BS, MD, FD	FS	Normal	Delay, slightly poor language performance	EIEE	1 month	OXC	
60	Female	c.4972C > T(p.P1658S)	Reported	*De novo*	2 months	7 years 2 months	FD	FS	Normal	Delay, walk at 2 years 8 months, speak at 9 months	EIEE	Non-remission		
61	Male	c.3956G > A(p.R1319Q)	Reported	*De novo*	2 months	6 years 3 months	FD, FS	FS	DFL	Delay, normal motor development, cannot speak	DEE	5 months	OXC	ASD
62	Male	c.2870C > A(p.T957N)	Novel	*De novo*	2 days	4 years 4 months	FD	FS	Normal	Normal	BIE	3 months	OXC	
63	Male	c.2558G > A(p.R853Q)	Reported	*De novo*	8 months	1 year 4 months	HPS	FS, SS	ACC	Delay, cannot control head and speak	WS	Non-remission		
64	Male	c.2995G > A(p.E999K)	Reported	*De novo*	1 day	1 year 3 months	BS, HPS, MFS, SS	FS, SS	Normal	Delay, cannot control head and speak	EIMFS	1 year	CBZ	
65	Male	c.4901G > T(p.G1634V)	Reported	*De novo*	1 days	3 months	BS, MFS, SE	FS, SS	ACC, ELV	Delay, cannot control head	EIMFS	Non-remission		Died at 3 months
66	Female	c.3631G > A(p.E1211K)	Reported	*De novo*	10 months	4 years 2 months	HPS, FD, SS, NCSE	FS, SS	Normal	Delay before seizure onset, cannot walk and speak	WS	2 years 2 months	VPA	
67	Female	c.4969C > T(p.L1657F)	Novel	*De novo*	6 days	2 years 8 months	HPS, GD, MD, FD, SS, FS	FS, SS	ACC, ELV, DFTL	Delay, cannot control head and speak	EIEE	Non-remission		
68	Male	c.4391C > T(p.T1464I)	Novel	*De novo*	2 days	2 years 2 months	FS, GD, MD, TS	FS, SS, TS	DWMM	Delay, control head at 2 years, cannot sit and speak	EIEE	1 year 3 months	VGB, PRP	
69	Female	c.1288G > A(p.E430K)	Novel	*De novo*	8 days	1 year 8 months	BS, HPS, MD, FS, SS	FS, SS, TSS	ACC, DWMM, DFTL, ELV	Delay, cannot control head and speak	OS → WS	Non-remission		
70	Male	c.707C > G(p.T236S)	Reported	*De novo*	20 days	6 months	BS, HPS, FS, TSS, SS	FS, SS, TSS	ACC	Delay, cannot control head and speak	OS → WS	Non-remission		
71	Male	c.5645G > A(p.R1882Q)	Reported	*De novo*	2 days	9 months	BS, HPS, GD, FS, SE, SS	FS, TSS	Normal	Delay, cannot control head and speak	OS → WS	Non-remission		
72	Male	c.4610T > C(p.I1537T)	Novel	Paternal	3 months	1 year 6 months	MD	FS	Normal	Normal	BFIE	4 months	LEV	

*MS, myoclonic seizure; FS, focal seizure; GTCS, generalized tonic clonic seizure; AtS, atonic seizure; SS, spasm; TSS, tonic spasm seizure; TS, tonic seizure; AS, absence seizure; FD, focal discharges; MD, multifocal discharges; BS, burst suppression; GD, generalized discharges; HPS, hypsarrhythmia; SE, status epilepticus; NCSE, nonconvulsive status epilepticus; DWMM, delayed white matter myelination; ELV, Enlargement of lateral ventricles; ACC, agenesis of corpus callosum; DFTL, dysplasia of frontotemporal lobes; DFL, dysplasia of frontal lobes; HA, hippocampal atrophy; BFNIE, benign familial neonatal-infantile epilepsy; BNE, benign neonatal epilepsy; BIE, benign infantile epilepsy; WS, West syndrome; EIEE, early infantile epileptic encephalopathy; OS, Ohtahara syndrome; DEE, developmental and epileptic encephalopathy. EIMFS, epilepsy of infancy with migrating focal seizures; FSs, febrile seizures; FSP, febrile seizures plus; DS, Dravet syndrome; KD, ketogenic diet; OXC, oxcarbazepine; LEV, levetiracetam; TPM, topiramate; VPA, valproate; PB, phenobarbital; CLZ, clonazepam; LTG, lamotrigine; KD, ketogenic diet; CBZ, carbamazepine; PRP, perampanel; VGB, vigabatrin; ASD, autistic spectrum disorder.*

### Clinical Phenotypes of Patients With *SCN2A* Variants

Among 72 patients with *SCN2A* variants, 50 are men, 22 are women. The seizure onset age was ranged from the first day of life to 2 years and 6 months. A total of 36 patients had seizure onset in neonates (50.0%, 36/72). A total of 18 patients had seizure onset between 1 and 6 months of age (25.0%, 18/72). A total of 11 patients had seizure onset between 7 month and 1 year of age (15.3%, 11/72). Seizure onset age was beyond 1 year in 7 patients (9.7%, 7/72). The seizure onset age of 5 patients with non-missense variants was between 11 months to 2 years and 6 months.

Focal seizures were observed in 65 patients(90.3%, 65/72), epileptic spasms in 38(52.8%, 38/72), tonic spasms in 15, myoclonic seizures in 5, tonic seizures in 4, generalized tonic-clonic seizures in 4, absence seizures in 3, and atonic seizures in 1, respectively. A total of 41 (41/72, 56.9%) patients presented 2 or more seizure types. Seizures manifested fever-sensitivity in 6 (8.3%, 6/72) patients (patient 9, 10, 20, 25, 50, and 55).

A total of 72 patients with *SCN2A* variants underwent video EEG. Interictal EEG abnormalities were heterogeneous, such as focal or multifocal epileptic discharges in 45 patients, hypsarrhythmia in 30, burst suppression in 18, and generalized discharges in 13, respectively. A total of 10 patients had normal interictal EEG. Seizures were recorded in 48 patients, such as focal seizures in 34 patients, epileptic spasms in 31, tonic spasms in 4, myoclonic seizures in 5, tonic seizures in 4, and absence seizures in 3, respectively.

**FIGURE 2:**
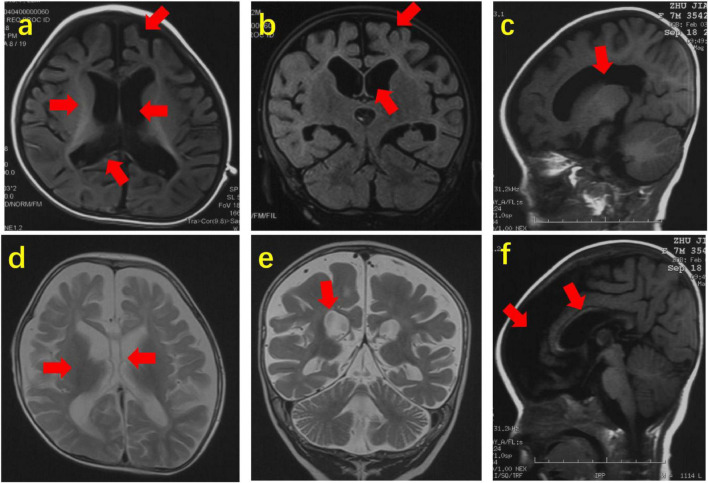
Abnormal brain MRI of 3 patients with *SCN2A* variants. Brain MRI of Patient 14 at the age of 22 months. **(a,b)**. Axial and coronal images (T1WI) showing agenesis of corpus callosum, delayed white matter myelination, dysplasia of frontotemporal lobes and enlargement of lateral ventricles. Brain MRI of Patient 29 at the age of 7 months. **(c,f)** Sagittal images (T1WI) showing enlargement of lateral ventricles, agenesis of corpus callosum and dysplasia of frontotemporal lobes. Brain MRI of Patient 39 at the age of 12 months. **(d,e)** Axial and coronal images (T2WI) showing agenesis of corpus callosum, delayed white matter myelination and enlargement of lateral ventricles. The arrow points to the lesion.

Brain MRI was performed in all 72 patients with *SCN2A* variants, which revealed abnormalities in 29 (29/72, 40.3%) patients. The abnormalities included dysplasia of frontal or frontotemporal lobes in 16, enlargement of the unilateral or bilateral lateral ventricle in 13, agenesis of the corpus callosum in 12, delayed white matter myelination in 8, and hippocampal atrophy in 2 patients, respectively (see Figure 2). The other 43 probands had normal brain MRI.

Among 72 patients with *SCN2A* variants, 57 patients (57/72, 79.2%) had varying degrees of developmental delay, and the other 15 patients had normal development. The 28 of 57 (28/57, 49.1%) patients with developmental delay cannot control head at last follow-up (median age: 2 years and 7 months; range: 3 months to 8 years and 2 months). All patients had epileptic spasms, burst suppression and hypsarrhythmia, abnormal MRI findings had developmental delay. Autism spectrum disorder (ASD) was diagnosed in 3 patients. All affected parents of the proband had normal development.

**FIGURE 3:**
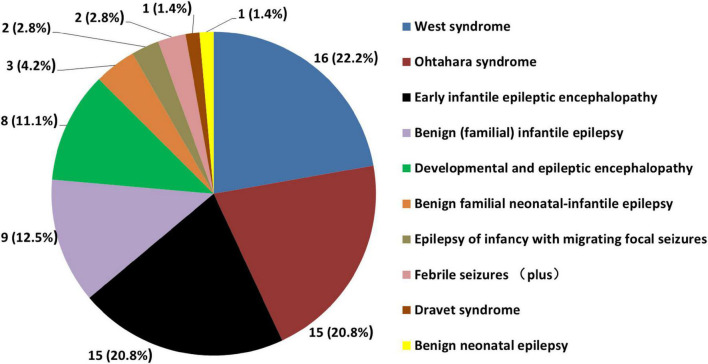
Distribution of 72 Chinese epilepsy patients with *SCN2A* variants according to phenotypes [*n* (%)].

In 72 patients, the phenotypes were diagnosed febrile seizures (plus) (*n* = 2), benign (familial) infantile epilepsy (*n* = 9), benign familial neonatal-infantile epilepsy (*n* = 3), benign neonatal epilepsy (*n* = 1), West syndrome (*n* = 16), Ohtahara syndrome (*n* = 15), epilepsy of infancy with migrating focal seizures (EIMFS) (*n* = 2), Dravet syndrome (*n* = 1), early infantile epileptic encephalopathy (EIEE) (*n* = 15), and unclassifiable developmental and epileptic encephalopathy (DEE) (*n* = 8) (Figure 3). A total of 16 (16/72, 22.2%) patients were initially diagnosed with West syndrome. A total of 15 patients (15/72, 20.8%) were diagnosed with Ohtahara syndrome at first, but 12 (12/15, 80%) of them evolved into West syndrome afterward. Both Patient 9 and Patient 10 were probands of generalized epilepsy with febrile seizures plus (GEFS+) families. Several paternal family members of Patient 9 had histories of febrile seizures in childhood. The Patient 10 was detected with maternal *SCN2A* variation I1663T which was inherited from his grandmother. His mother had 2 febrile seizures in early childhood. However, his maternal grandmother had no history of seizures. The Patient 48 was diagnosed with intractable West syndrome, recurrent *SCN2A* variation R853Q was detected. His mosaic mother had several seizures before 1 year of age. Her seizures were self-limited without using any ASM therapy.

### Genotype–Phenotype Correlation

Of the 59 *SCN2A* variants, 31 were located in transmembrane regions, while the other 28 were in non-transmembrane regions. Carriers of 2 recurrent variants (R1319Q and V261M) included both patients with normal development (Patient 57 and Patient 49) and patients with developmental delay (Patient 61, Patient 12, and Patient 30). A total of 13 variants were only detected in patients with normal development, 9 (9/13, 69.2%) of them were located in non-transmembrane regions, and the remaining 4 were located in transmembrane regions which accounting for 12.9% (4/31) of all transmembrane region variations. A total of 44 variants were only detected in patients with developmental delay, 26 (26/44, 59.1%) were located in transmembrane regions, accounting for 83.9% (26/31) of transmembrane region variants, and 18 were located in non-transmembrane regions (see Figure 1). The 10 of 12 (83.3%) patients with inherited *SCN2A* variants had normal intelligence; however, the other 2 (2/12, 16.7%) patients had a developmental delay (Patient 10 and 48). Among 60 patients with *de novo SCN2A* variants, 55 had development delay (91.7%, 55/60), and the remaining 5 patients had normal development.

### Seizure Treatment and Prognosis

At the last follow-up (median age: 4 years and 4 months; range: 3 months to 13 years), 39 (54.2%, 39/72) patients were seizure-free at a median age of 8 months (range: 1 month to 5 years 4 months of age), the remaining 33 patients still had refractory seizures (median age: 2 years 8 months; range: 3 months to 13 years). Among 39 patients with seizure freedom, 2 patients who were diagnosed with benign familial infantile epilepsy did not use any ASM therapy, 21 (21/39, 53.8%) patients used monotherapy, 11 used two-drug treatment, and 5 used polytherapy. All 33 patients with uncontrolled seizures have tried at least 2 ASM therapies. All 15 patients had normal development were seizure-free. Of the 29 children with abnormal brain MRI, 23 (23/29, 79.3%) patients still had seizures at the last follow-up, and only 6 had seizure freedom.

**FIGURE 4:**
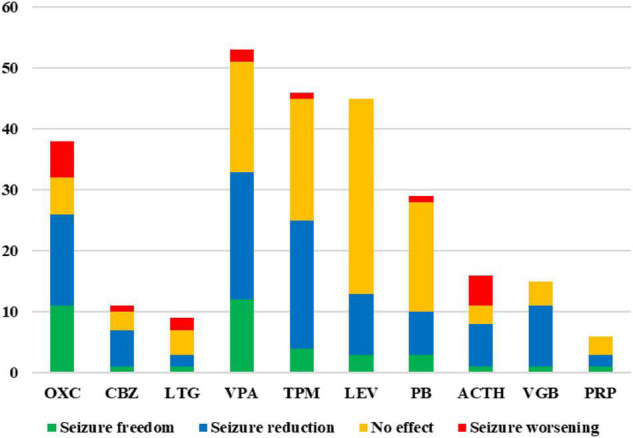
Treatment effects of anti-seizure medication (ASM) therapies in epilepsy patients with *SCN2A* variants. Number of treated patients and their seizure outcome (green = seizure freedom; blue = seizure reduction; orange = no effect; red = seizure worsening) that have been treated with different ASM therapies. Only effects of ASM therapies that at least one patient achieved seizure free are shown. OXC, oxcarbazepine; CBZ, carbamazepine; LTG, lamotrigine; VPA, valproate; TPM, topiramate; LEV, levetiracetam; PB, phenobarbital; VGB, vigabatrin; PRP, perampanel.

At least one patient in the study experienced seizure control after treatment with SCBs such as oxcarbazepine, carbamazepine, lamotrigine, and other ASM therapy like valproate, topiramate, levetiracetam, phenobarbital, ACTH, vigabatrin, and perampanel. The effect of these ASM therapies is shown in Figure 4. No patient experienced seizure control after using phenytoin, zonisamide, lacosamide, clonazepam, nitrazepam, clobazam, cannabidiol, ketogenic diet, and vagus nerve stimulation.

Oxcarbazepine has been used in 38 patients, seizure freedom, seizure reduction, no effect, and seizure worsening were observed in 11 (11/38, 28.9%), 15 (15/38, 39.5%), 6 (6/38, 15.8%), and 6 (6/38, 15.8%) patients, respectively. Among those 38 patients, 35 patients had seizure onset age < 3 months, 6 patients had seizure onset age between 4 months and 1 year of age, and the other 3 patients had seizure onset age > 1 year. For 29 patients with seizure onset age < 3 months, seizure freedom, seizure reduction, no effect, and seizure worsening were observed in 8 (8/29, 27.6%), 13 (13/29, 44.8%), 5 (5/29, 17.2%), and 3 (3/29, 10.3%) patients (patients 20, 25, and 37), respectively. For 6 patients with seizure onset age between 4 months and 1 year of age, seizure freedom, seizure reduction, and no effect were observed in 3, 2, and 1 patient, respectively, no patients experienced seizure worsening. All 3 patients with seizure onset age > 1 year had seizure exacerbation caused by oxcarbazepine (Patients 27, 47, and 50). The Patient 10 was diagnosed with Dravet syndrome and his seizure was controlled after the addition of oxcarbazepine at the age of 5 years old and had no relapse for nearly 2 years. The effects of oxcarbazepine in patients with different *SCN2A* variants have been presented in Figure 1. Carbamazepine has been used in 11 patients, seizure freedom, seizure reduction, no effect, and seizure worsening were observed in 1 (1/11, 9.1%), 6 (6/11, 54.5%), 3 (3/11, 27.3%), and 1 (1/11, 9.1%) patients, respectively. Lamotrigine has been used in 9 patients, seizure freedom, seizure reduction, no effect, and seizure worsening were observed in 1 (1/9, 11.1%), 2(2/9, 22.2%), 4(4/9, 44.4%), and 2 (2/9, 22.2%) patients, respectively.

Valproate has been used in 53 patients with *SCN2A* variants, seizure freedom, seizure reduction, no effect, and seizure worsening were observed in 12 (12/53, 22.6%), 21 (21/53, 39.6%), 18 (18/53, 34.0%), and 2 (2/53, 3.8%) patients, respectively. Seizures were controlled by topiramate, levetiracetam, and phenobarbital in 4 (4/46, 8.7%), 3 (3/45, 6.7%), and 3 (3/29, 10.3%) patients, respectively. One patient was seizure free after taking ACTH, vigabatrin, and perampanel, respectively. No patient had seizure exacerbation caused by levetiracetam, vigabatrin, and perampanel. Seizure worsening caused by ACTH was observed in 5 (5/16, 31.3%) patients.

Five (5/72, 6.9%) patients died at the age of 3 months to 4 years and 3 months (Patients 11, 14, 15, 29, and 65). All those 5 patients started seizures in neonate. Four patients were initially diagnosed with Ohtahara syndrome, and the other patient diagnosed with EIMFS. Three of them (Patients 14, 29, and 65) manifested intractable seizures with no effect to multiple ASM therapies. The causes of those 3 patients were unknown. Both Patients 11 and 15 were seizure free at the age of 8 months, and suffered possible sudden unexpected death in epilepsy (SUDEP).

## Discussion

*SCN2A* gene is located on chromosome 2q24.3. The gene which contains 26 exons encodes the α2 subunit of the voltage-gated sodium channel (Nav1.2). Nav1.2 is mainly expressed in the initial part of excitatory neuron axons and unmyelinated axons. The protein is widely distributed in the cortex, hippocampus, striatum, and midbrain. Variations in *SCN2A* gene are associated with a spectrum of neurodevelopmental and epileptic disorders, such as epilepsy, intellectual disability, ASD, schizophrenia, and periodic ataxia, presenting an autosomal dominant inheritance (Carroll et al., 2016; Yokoi et al., 2018; Long et al., 2019; Schwarz et al., 2019; Suddaby et al., 2019; Epifanio et al., 2021). In recent years, a lot of *SCN2A* variants have been reported. The variation types include missense variation, in-frame deletion or insertion variation, non-sense variation, frameshift variation, and splice site variation. It has been reported that missense variation was the most common variation type of *SCN2A* variants (Wolff et al., 2017). In this study, we have found 59 *SCN2A* variants in 72 Chinese epilepsy patients, and 22 of them are novel variants. The *SCN2A* variants detected in our study show no hotspot and more than 90% of the variants are missense variants. Other variation types such as in-frame deletion or insertion variant, non-sense variant, and frameshift variant were also presented in our study, but the percentage is small. In our cohort, more than 80% of patients had *de novo SCN2A* variants.

Sugawara et al. (2001) firstly reported *SCN2A* missense variant R187W in a Japanese family with GEFS+. The affected patients in this family showed febrile seizures and focal epilepsy. Heron et al. (2002) reported that *SCN2A* gene was the major causative gene of benign familial neonatal-infantile epilepsy. At first, some researchers believed that missense variations tend to result in benign epilepsy, whereas truncation variations lead to severe and intractable epilepsy (Yamakawa, 2006). With the wide application of next-generation sequencing in clinical practice, *SCN2A* variants have been reported in severe early onset epileptic encephalopathy and most of them were *de novo* variants.

Most patients with *SCN2A* variants start seizures in early childhood. Wolff et al. (2017) reported that about half of patients with *SCN2A* variants had seizure onset in the neonate. In our study, half of the patients started seizures during the neonatal period. Nearly 80% of patients started seizures within 6 months of age. It suggests that seizures caused by *SCN2A* variants tend to start in early infancy. The seizure types of patients with *SCN2A* variants are varied. In our cohort, the most common seizure types were focal seizures and epileptic spasms, observed in about 90% and 50% of patients, respectively. Other seizure types, such as tonic spasm, myoclonic seizures, atonic seizures, tonic seizures, clonic seizures, generalized tonic-clonic seizures, and absence seizures were also observed in some patients. Those seizure types were relatively rare but all of them have been reported in the literature. In our study, the abnormal interictal EEG of epilepsy patients with *SCN2A* variants such as focal or multifocal epileptic discharges, hypsarrhythmia, burst suppression, and generalized discharges. About 40% of patients in this study had brain MRI abnormalities, such as dysplasia of the frontal or frontotemporal lobes, enlargement of unilateral or bilateral lateral ventricle, agenesis of the corpus callosum, delayed white matter myelination, and hippocampal atrophy. The dysplasia of frontal or frontotemporal lobes is a common defect in our patients with brain MRI abnormalities. The abnormal neuroimages mainly indicated cerebral dysplasia. Nearly 80% of patients had developmental delays, and about half of them cannot control their heads at a median age of 2 years and 7 months.

Wolff et al. (2017) reported 66 families or sporadic cases with *SCN2A* variants which were collected by a multicenter study that participated by 74 clinical or research institutions. The phenotypes reported in the multicenter study included benign (familial) neonatal/infantile epilepsy, Ohtahara syndrome, EIMFS, encephalopathy with early infantile-onset epilepsy, West syndrome, myoclonic-atonic epilepsy, Lennox–Gastaut syndrome, epileptic encephalopathy with infantile/childhood-onset epilepsy, intellectual disability, and/or autism without epilepsy. Most of the patients were diagnosed with epilepsy. In this study, we analyzed the epilepsy phenotypes of patients with *SCN2A* variants. The oldest child in our group at the last follow-up was 13 years old. Some of the patients in this study were diagnosed with epilepsy at our clinical center at an early stage of life and were confirmed with *SCN2A* variants in recent years. The common epilepsy phenotypes of patients with *SCN2A* variants include benign epilepsy in the first year of life, Ohtahara syndrome, West syndrome, and EIEE. Those epilepsy phenotypes account for more than 80% of this cohort. Most of the patients who were initially diagnosed with Ohtahara syndrome evolved into infantile spasms. Other rare epilepsy phenotypes include febrile seizures (plus), EIMFS, and Dravet syndrome in our study. Wolff et al. (2017) did not report any patient with Dravet syndrome, febrile seizures, or febrile seizures plus. In addition, only 6 patients (8.3%, 6/72) in our study had fever-sensitive seizures, indicating that fever sensitivity is a rare feature of epilepsy patients with *SCN2A* variants. All those 8 patients diagnosed with unclassifiable DEE had a developmental delay before seizure onset and developmental regression after seizure onset and cannot be diagnosed with any known epilepsy syndrome. In this study, some patients with developmental delay showed little improvement after seizure control, which further suggests that the variation itself has a significant impact on brain development. The mother of Patient 48 carried a mosaic *SCN2A* variant R853Q with a ratio of about 20% in the peripheral blood. R853Q is a recurrent variation that has been reported repeatedly in the literature (Ganguly et al., 2021). Both the Patient 48 and two other patients with the same variant in our study, as well as patients with the same variant reported in the literature, were diagnosed with West syndrome. However, the mother of Patient 48 had self-limited seizures before 1 year of age and normal development. It indicates that the phenotype severity caused by *SCN2A* variants is related to the dose of variation.

At the last follow-up, about half of the patients (54.2%, 39/72) were seizure-free at the median age of 8 months. Few of them were self-limited. Liao et al. (2010) found that *SCN2A* had high expression in the initial segment of the axon of hippocampal neuron of a mouse during 5–15 days after birth, and the function was gradually replaced by the protein encoded by *SCN8A*. It speculated that this may be the reason why benign epilepsy due to *SCN2A* variants could be self-limited with age. About half of the patients with seizure control were treated with monotherapy, while the rest were treated with 2 or 3 drugs. The seizures were not controlled in nearly half of the patients, and the median age of these patients at the last follow-up was 2 years and 8 months, with the oldest being 13 years. All those patients presented with refractory epilepsy. Seizures were not controlled in all patients with brain MRI abnormalities in this study.

It has been suggested that SCBs are effective drugs in the treatment of epilepsy of patients with *SCN2A* variation (Reif et al., 2017). More than half of the patients in our study had used SCBs. Probably because it comes in liquid form, oxcarbazepine was the most commonly used SCBs in our cohort which has been used in 39 patients. Although oxcarbazepine was indeed the most effective ASM therapy in our study, the rate of seizure control was still less than 30%. It has been reported (Wolff et al., 2017) that, patients with seizure onset age less than 3 months always carry *SCN2A* variants that cause the gain of function and SCBs were often effective for seizures. However, those patients had a seizure onset age later than 3 months, *SCN2A* variation often causes loss of function and SCBs worsen the seizure. Brunklaus et al. (2020) also reported that individuals with gain-of-function *SCN2A*/3A/8A most frequently present with early-onset epilepsy (<3 months), and have a good response to SCBs, which is not completely consistent with our results. In this study, about 27% of patients with seizure onset age <3 months had seizures controlled by oxcarbazepine, but another 3 patients had seizure exacerbation. Half of the patients with onset age from 4 months to 1 year had seizure control after administration of oxcarbazepine and no patient presented seizure exacerbation. All 3 patients with onset age > 1 year had seizure exacerbation due to oxcarbazepine. *SCN2A* gain-of-function has recently been recognized as a cause of early infantile-onset epileptic encephalopathies, whereas loss-of-function *SCN2A* variations often cause ASD or intellectual disability with later-onset mild epilepsy or without epilepsy (Yamakawa, 2006; Ben-Shalom et al., 2017; Wolff et al., 2017). Based on the results of our study and those in the literature, SCBs are not recommended for patients with seizure onset age > 1 year, while SCBs can be tried for patients with seizure onset age < 1 year, but the possibility of seizure exacerbation still needs to be warned. Phenotypes caused by *SCN2A* variation are associated with underlying functional changes caused by the variants. Although the rate of seizure control was low, in this study, seizure control was observed in one patient by carbamazepine and lamotrigine, respectively. Since a few cases of Dravet syndrome caused by *SCN2A* variants has been reported (Wang et al., 2012), the effect of SCBs for those patients has not been reported. In our study, Patient 10 was diagnosed with Dravet syndrome, he was seizure free after adding oxcarbazepine at the age of 5 years old, suggesting that Dravet syndrome is caused by *SCN2A* variant. It is different from that Dravet syndrome caused by *SCN1A* variation is mostly not responsive to or might even be exacerbated by SCBs. Although *SCN2A* and *SCN1A* are both sodium channel genes, the underlying pathogenesis of Dravet syndrome caused by *SCN2A* variation may be different from that of *SCN1A*.

Besides of SCBs, valproate was also the effective ASM therapy in this study, with a seizure control rate of about 22%, slightly lower than oxcarbazepine. In addition, the proportion of exacerbations caused by valproate was lower than that caused by oxcarbazepine. In this study, a large number of patients had used topiramate, levetiracetam, and phenobarbital, and seizure control was achieved in some patients by each of those drugs, although the rates of seizure control were relatively low. In addition, although there were fewer patients who had used ACTH, vigabatrin, and perampanel, there was one case of seizure control for each drug, respectively. ACTH is the preferred drug for the treatment of West syndrome, but nearly one-third of the patients in this study experienced increased seizure frequency after the use of ACTH. The specific mechanism of exacerbation of seizures needs to be further studied. No seizure exacerbation occurred after taking levetiracetam, perampanel, or vigabatrin.

In this study, 5 children died, accounting for about 7% (5/72) of the cases in this group. All 5 patients started seizures during the neonatal period and were diagnosed with severe epileptic syndromes such as Ohtahara syndrome or EIMFS. Two of the patients were seizure-free and the possible cause of death was SUDEP, while the other 3 patients still had frequent seizures and the exact cause of death was unknown. The underlying pathophysiology of SUDEP remains unclear. SUDEP cannot be predicted in advance, because the complete underlying pathophysiology of the phenomenon is likely multifactorial and prognostic biomarkers were still not found (Goldman et al., 2016; Sahly et al., 2022). Our study indicates that frequent seizures and *SCN2A* variations themselves may be important factors leading to death in these patients.

Phenotypes caused by *SCN2A* variants are heterogeneous. It has been reported that phenotypic variability from benign infantile epilepsy to Ohtahara syndrome was observed in 3 affected individuals of a family with *SCN2A* variation (Syrbe et al., 2016). In our study, both benign epilepsy with normal development and epileptic encephalopathy with developmental delay were observed in unrelated patients carrying variants R1319Q and V261M. This was also confirmed by the different phenotypes in the family of Patient 48, ranging from normal to Dravet syndrome in the carriers of the same *SCN2A* variant.

More than 80% of patients with inherited *SCN2A* variants had a benign outcome in our study. However, more than 90% of patients with *de novo SCN2A* variants showed developmental delay, which was identical to the reported studies (Wolff et al., 2017). In this study, the seizure onset age of patients with non-missense *SCN2A* variants was late, and the seizure onset age was after 11 months, which was consistent with the literature reports (Lauxmann et al., 2018). It may be most non-missense variations will lead to loss-of-function, and the early manifestations of such functional changes are mostly developmental delay or autism, while epilepsy often begins in late infancy or early childhood (Begemann et al., 2019). The number of *SCN2A* variants located in transmembrane regions was similar to that in non-transmembrane regions in our study. Only 13 *SCN2A* variants merely in patients with normal development, and about 70% of them were located in non-transmembrane regions. However, more than 80% of variants located in transmembrane regions were related to patients with developmental delay. This may be due to *SCN2A* variants in transmembrane regions having a greater effect on protein function than those in non-transmembrane regions.

*SCN2A* is a common causative gene of genetic epilepsy in children. Patients with *SCN2A de novo* variation usually have a poor prognosis lacking precise treatment. In this study, epilepsy patients with *SCN2A* variants from 2 pediatric clinical centers in China were studied. Multi-center, large-sample, and prospective studies are needed to further analyze the genotype–phenotype correlations of *SCN2A*-related epilepsy for precise medicine.

## Data Availability Statement

The data presented in this study are available through Clinvar (http://www.clinvar.com/), with the following accession numbers SCV002099454 – SCV002099517. Further inquiry can be directed to the corresponding author.

## Ethics Statement

The studies involving human participants were reviewed and approved by Peking University First Hospital and Shenzhen Children’s hospital. Written informed consent to participate in this study was provided by the participants’ legal guardian/next of kin.

## Author Contributions

YZ and JXL contributed to the design and implementation of the research. ZY and YJ were involved in supervised the work. JD and QZ were responsible for assessing the pathogenicity of variants. XN, YC, DW, JZ, JC, XY, and JLL were responsible for follow-up of the patients. QZ and YY contributed to the analysis of the results and to the writing of the manuscript. All authors contributed to the article and approved the submitted version.

## Conflict of Interest

The authors declare that the research was conducted in the absence of any commercial or financial relationships that could be construed as a potential conflict of interest.

## Publisher’s Note

All claims expressed in this article are solely those of the authors and do not necessarily represent those of their affiliated organizations, or those of the publisher, the editors and the reviewers. Any product that may be evaluated in this article, or claim that may be made by its manufacturer, is not guaranteed or endorsed by the publisher.
